# Silica nanoparticles as advanced platforms for nucleic acid delivery

**DOI:** 10.1016/j.mtbio.2026.102921

**Published:** 2026-02-10

**Authors:** Mónica L. Fanarraga, Lorena García Hevia

**Affiliations:** a The Nanomedicine Group, Valdecilla Health Research Institute (IDIVAL), Faculty of Medicine, Universidad de Cantabria, Santander, 39011, Spain; b CINBIO, Department of Biochemistry, Genetics and Immunology, Universidade de Vigo. Galicia Sur Institute of Health Research (IISGS), Vigo, 36310, Spain

**Keywords:** Silica nanoparticles, Gene delivery, Stimuli-responsive nanocarriers, Hybrid nanoplatforms, Biodegradability

## Abstract

Nucleic acid therapeutics, including siRNA, mRNA, plasmid DNA, and CRISPR/Cas systems, have demonstrated remarkable potential but continue to face translational barriers related to systemic instability, immune activation, and inefficient intracellular delivery. Conventional lipid and polymeric carriers, although clinically validated, often lack the structural resilience and versatility required for large or complex cargos. Silica-based nanoparticles, particularly mesoporous silica nanoparticles, provide a distinctive combination of mechanical rigidity, tunable porosity, and abundant surface chemistry that enables robust encapsulation, protection, and controlled release of diverse nucleic acids.

This review adopts a problem-driven perspective, analyzing how specific nanoarchitectural designs, surface functionalizations, and ligand-mediated targeting strategies address key limitations in nucleic acid delivery. Emphasis is placed on overcoming systemic barriers such as premature degradation, immune recognition, and restricted biodistribution, as well as intracellular challenges including endosomal escape and nuclear access. Hybrid and biomimetic silica platforms are highlighted for their capacity to integrate combinatorial and theranostic functionalities, expanding the therapeutic scope toward complex payloads and multifunctional formulations.

By linking synthesis approaches with translational requirements, an integrated roadmap is proposed that positions silica nanocarriers as advanced platforms for next-generation gene therapy. The evidence underscores the potential of silica architectures to combine structural durability with versatile chemical adaptability, thereby enabling safe, efficient, and clinically relevant delivery of nucleic acids.

## Graphical abstract

**Figure:**
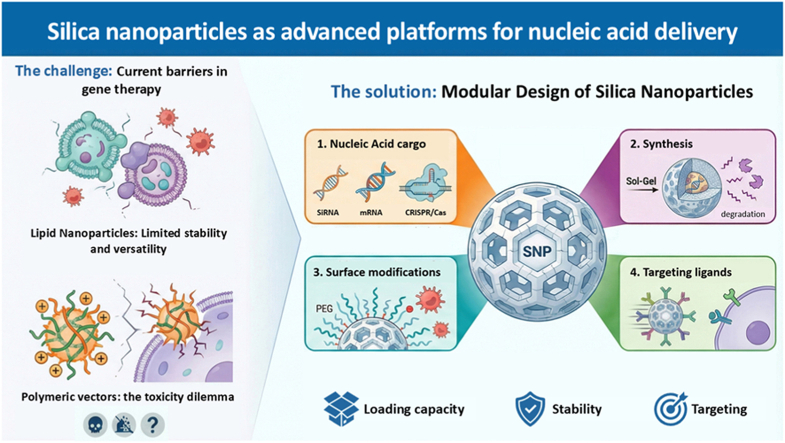

## 1 Introduction

Despite advances in nucleic acid therapeutics, including plasmid DNA, small interfering RNA (siRNA), messenger RNA (mRNA), and CRISPR/Cas systems, many candidates fail to translate into clinically approved therapies. Delivery inefficiencies, systemic instability, and immunogenicity remain frequent limiting factors [[1], [2], [3], [4], [5]]. Lipid nanoparticles (LNPs), while central to the success of mRNA vaccines, exhibit limited versatility beyond hepatic or intramuscular delivery [[6], [7], [8]]. Their physicochemical fragility predisposes them to aggregation or premature release in blood plasma [9,10]. Moreover, lipid components can activate innate immunity pathways, such as Toll-like receptors and complement cascades, compromising repeat dosing and safety [11,12].

Beyond LNPs, a wide range of delivery platforms, including polymeric carriers, inorganic nanoparticles, protein-based vectors and extracellular vesicles, have been explored to improve nucleic acid stability, targeting and intracellular trafficking, with varying degrees of preclinical success. Nevertheless, most systems face challenges related to structural stability, reproducibility, targeting efficiency and regulatory translation, underscoring the need for alternative carrier architectures that combine robustness, tunability and biological compatibility.

Polymeric carriers, although highly tunable, face the polycation dilemma: they require sufficient positive charge to complex nucleic acids effectively, yet excessive charge density induces cytotoxicity, membrane disruption, and metabolic stress [[13], [14], [15], [16]]. Additionally, conventional soft-matter vectors are often poorly suited to large or multicomponent cargos, such as plasmid DNA, self-amplifying mRNA, or CRISPR/Cas ribonucleoprotein complexes, due to limited internal volume and mechanical stability for efficient encapsulation and controlled release [17,18].

Collectively, these limitations constitute a persistent translational bottleneck. Addressing this challenge requires delivery platforms that combine molecular-level tunability with mechanical robustness and biocompatibility. Silicon shares chemical similarities with carbon, and its oxide form (SiO_2_) is widely recognized for biocompatibility and versatility. In this context, silica-based nanoparticles (SNPs) represent a broad class of inorganic nanocarriers, among which mesoporous silica nanoparticles (MSNs) constitute a structurally defined subclass characterized by ordered mesoporosity. MSNs combine mechanical rigidity, tunable porosity, and abundant surface chemistry [[19], [20], [21]]. Unlike purely organic carriers, SNPs integrate structural durability with versatile chemical functionality, positioning them as promising platforms to overcome the major barriers in gene therapy.

Within the broader class of silica-based nanoparticles, it is important to distinguish between MSNs and colloidal silica, as these platforms exhibit distinct structural features, degradation behaviors and delivery profiles. MSNs are characterized by ordered mesoporosity and are typically synthesized using structure-directing agents, enabling high loading capacity and precise control over release kinetics [22], particularly advantageous for siRNA, mRNA and multicomponent cargos [23]. In contrast, colloidal silica consists of non-porous or weakly porous nanoparticles produced via sol–gel routes, displaying gradual dissolution under physiological conditions and enhanced stability in acidic environments [24]. These properties make colloidal silica especially suitable for protecting large or sensitive nucleic acids during intracellular trafficking, including DNA incorporated directly into the silica condensation mixture [[25], [26], [27]], and for applications requiring controlled biodegradability [28]. Silica nanomaterials exhibit well-defined morphologies and tunable structures, including adjustable particles and pore sizes, large specific surface area, high mechanical and thermal stability, low toxicity, and excellent biocompatibility. Furthermore, silica particles can be easily functionalized with diverse ligands, enabling targeted delivery to specific cell types. Silica shells are frequently used to impart these advantageous properties to a wide range of nanomaterials. Mesoporous silica materials have demonstrated favorable biocompatibility both *in vitro* and *in vivo* when particle size, porosity, and surface charge are properly controlled [[29], [30], [31]]. Studies on calcined MSNs have confirmed cytocompatibility with human and animal cells, including blood-derived lines [32].

At the same time, surface silanol density and residual structure-directing agents can modulate cellular responses, underscoring the importance of surface engineering and thorough template removal for safe biomedical use [[33], [34], [35]]. The silica framework also enables precise control over morphology and pore architecture. Mesoporous, hollow, or “rattle-type” configurations can accommodate nucleic acid cargos of various sizes, from siRNA to bulky CRISPR/Cas complexes [[36], [37], [38], [39]]. Surface silanols serve as reactive anchors for polymers, ligands, and stimuli-responsive gatekeepers, while PEGylation or zwitterionic coatings enhance colloidal stability and reduce nonspecific protein adsorption [23,33,40].

Although MSNs remain the most widely studied silica-based carriers, recent research has demonstrated that colloidal silica, a non-porous or weakly porous form within the broader class of SNPs, can also serve as an effective platform for gene delivery, largely due to its unique physicochemical properties. Unlike MSNs, colloidal silica exhibits controlled biodegradability: it is stable in organic solvents but gradually dissolves in aqueous environments and more rapidly under physiological conditions (pH 7.4), forming silicic acid that is safely excreted *in vivo* [41]. This dissolution behavior ensures biocompatibility and enables controlled cargo release after cellular uptake. Moreover, colloidal silica is highly stable at acidic pH (5–6), which protects genetic material during endo-lysosomal trafficking—a critical advantage for intracellular delivery.

Beyond its biodegradability, colloidal silica offers exceptional thermal and chemical stability, shielding nucleic acids from DNases, oxidative stress, and even extreme temperatures (>100 °C) [[25], [26], [27]]. Its modular design allows encapsulation of large genetic cargos without size restrictions, including multi-layer architectures for sequential or time-controlled gene expression, mimicking viral cascades. These systems are scalable, reproducible, and cost-effective, facilitating large-scale production and transport, key requirements for personalized therapeutics.

**Fig. 1:**
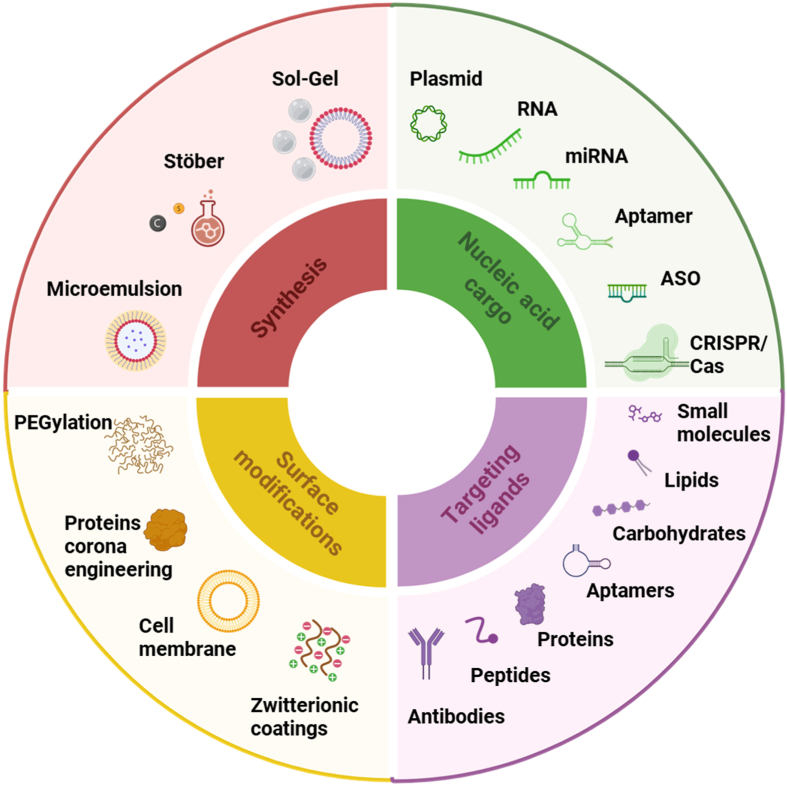
**Conceptual design map for silica-based nucleic acid delivery systems.** The diagram illustrates the four fundamental design domains that govern the engineering of silica nanoparticles for gene therapeutics: (green) nucleic acid cargo type which defines pore size, loading strategy and release requirements; (purple) targeting ligands, used to enhance receptor-mediated uptake and precision biodistribution; (yellow) surface modification, to modulate biological identity, colloidal stability and immune evasion; and (red) synthesis approach which determines particle morphology, porosity and framework structure. Surface modifications and targeting ligands are represented as distinct but complementary design layers.

Unlike many lipid or polymeric systems, SNPs preserve structural integrity under physiological stress, resisting aggregation, osmotic pressure, and enzymatic degradation, supporting reproducible pharmacokinetics and long-term formulation stability, key prerequisites for successful clinical translation. A unifying view of the design space for silica-based nucleic acid delivery, linking synthetic routes, cargo type, surface engineering and targeting ligands, is summarized in Fig. 1.

While previous reviews have summarized the applications of mesoporous silica nanoparticles for gene delivery, they largely adopt a descriptive, method-based structure. In contrast, this review follows a problem-driven perspective, focusing on how specific engineering choices in silica nanoparticle design address key translational barriers, particularly stability, immune evasion, intracellular release and delivery of large cargos such as mRNA and CRISPR/Cas systems. Emphasis is placed on advanced payloads, combinatorial and theranostic formulations, and hybrid silica-based platforms, providing an integrated roadmap from nanoarchitectural design to clinical translation.

### 1.1 Therapeutic nucleic acids cargos: implications for silica nanocarrier design

The structural and interfacial design of silica-based nanocarriers is intrinsically dictated by the characteristics of nucleic acid cargo. Although all gene therapeutics share an overall negative charge, they differ substantially in size, rigidity, intracellular site of action, and susceptibility to degradation. These parameters determine the required pore architecture, loading strategy, release mechanism, and the need for protective or targeting functionalities.

Short oligonucleotides such as siRNA (∼21–23 bp) and miRNA mimics exhibit moderate sensitivity to nucleases and primarily act in the cytosol via the RNA-induced silencing complex [42]. Their relatively small size facilitates loading into mesopores (typically 2–5 nm), often via electrostatic complexation with polycationic coatings [[43], [44], [45]]. Antisense oligonucleotides (ASOs) share similar molecular size but require sustained intracellular availability to modulate RNA splicing or inhibit translation, making kinetics release a key design aspect [46].

mRNA (300–500 kDa), on the other hand, presents greater structural flexibility but is highly susceptible to hydrolysis and enzymatic degradation. For successful translation, mRNA requires cytosolic localization, necessitating robust protection during systemic circulation until efficient endosomal escape is achieved. Accordingly, MSN formulations for mRNA typically employ enlarged pore apertures (≥10 nm) combined with redox-responsive or degradable frameworks to synchronize release with intracellular triggers [[47], [48], [49]].

Plasmid DNA (typically ∼3–20 kb) requires nuclear access following cytosolic delivery and demands substantial volumetric loading capacity along with extended protection and controlled release. Hollow or large-pore MSNs are particularly suited for plasmid delivery, often combined with surface-mediated complexation or hybrid lipid coatings to facilitate trafficking [23,50,51]. Recent studies have shown that directly embedding plasmid DNA within a colloidal silica matrix via one-pot synthesis can markedly improve its stability during storage and under harsh physicochemical or biological conditions, while retaining efficient transfection [26]. Related soluble silica platforms have also been used to encapsulate therapeutic oligonucleotides as prodrugs for targeted delivery in the tumor microenvironment, further underscoring the potential of silica architectures for robust, long-term DNA and DNA-based cargo delivery [25].

CRISPR/Cas ribonucleoproteins (RNPs, ∼160 kDa) represent a special case due to their mixed macromolecular composition (protein and RNA). Their efficient delivery requires preservation of conformational integrity, avoidance of lysosomal degradation and prompt cytoplasmic release for genome editing [38,52,53]. Hybrid architectures such as lipid-coated MSNs (LC-MSNs) or spiky silica systems have demonstrated improved intracellular trafficking for RNPs [36].

Emerging nucleic acid modalities, such as aptamers, self-amplifying RNA, circular RNA, therapeutic oligonucleotide prodrugs [25], expand the functional scope of silica carriers, offering opportunities for dual targeting, extended expression profiles, and combined therapeutic/diagnostic responses [54].

## 2 Engineering the silica nanoparticles shells to overcome systemic delivery barriers

Silica nanoparticles, particularly MSNs, are among the most versatile and widely studied nanomaterials in nanomedicine [34,55,56]. Their appeal stems from a highly tunable architecture, large surface area, adjustable pore size, and controllable morphology that together enables substantial cargo loading and sustained or stimuli-responsive release of therapeutic nucleic acids [57,58]. Well-established synthesis methods, such as the sol-gel and Stöber processes, allow precise control over these physicochemical parameters, ensuring reproducibility and predictable interactions with biological systems [23,34]. Since their introduction in the early 1990s [21], MSNs have evolved into adaptative, anisotropic, and multidomain architectures designed to overcome systemic barriers such as rapid clearance, poor tissue penetration, and heterogeneous tumor microenvironments [59,60]. These structural innovations have significantly improved their performance in nucleic acid delivery applications.

### 2.1 Stabilizing the cargo and evading the immune system

#### 2.1.1 Structural architectures for protection

The protective efficiency of MSNs is closely linked to the internal architecture governing encapsulation and release dynamics. Mesoporous, hollow, and core–shell silica nanostructures enable encapsulation of nucleic acids within confined cavities, shielding them from enzymatic degradation and oxidative stress [61,62]. The rigid silica matrix limits premature leakage from the pore network, whereas pore-entrance engineering and interfacial gating dictate diffusional selectivity: small molecules (sub-nanometer to ∼1–2 nm hydrodynamic diameter) permeate mesopores, while nucleases are effectively excluded either when pore mouths are narrower than their hydrodynamic size or when entrances are sealed by polymer/ligand gates or supported lipid bilayers that remain closed until an intracellular trigger is encountered [23,63,64]. This architecture-driven control of access, size-restricted entrances complemented by stimulus-responsive gates, underpins the balance between cargo protection and on-demand release elaborated in subsequent sections.

Recent studies have shown that direct embedding DNA during synthesis significantly enhances resistance to serum nucleases versus simple surface adsorption approaches [25,26]. In addition, hybrid designs, such as polymer or lipid-coated MSNs, combine the mechanical robustness of silica with the adaptability of organic components, resulting in enhanced protection and tunable release profiles [36,38,65]. Biodegradable silica nanoparticles have also been shown to efficiently deliver linear DNA constructs while undergoing controlled degradation into excretable species [27]. Furthermore, a one-pot synthesis approach has yielded compact DNA–silica particles with exceptional preservation of genetic material, offering a scalable and protective platform for gene delivery [26].

While internal architecture is essential for effective cargo encapsulation, protection, and release control, does not fully determine the *in vivo* performance of MSN-based delivery systems. Once structural design parameters are established, interactions with biological environments are predominantly governed by surface properties, which dictate protein adsorption, immune recognition, and circulation behavior. Accordingly, surface functionalization represents a complementary and equally critical design layer, as discussed in the following section.

Multiple synthesis strategies have been developed to optimize MSN–nucleic acid complexes, each with distinct trade-offs in terms of loading efficiency, structural integrity, and biological performance. Table 1 summarizes representative approaches.

**Table 1:** Representative synthesis strategies for nucleic acid–mesoporous silica nanoparticle (MSN) complexes.

Synthesis methods		Description	Advantages	Disadvantages	Key application in nucleic acid delivery	Ref.
Classical Stöber and derivatives	Modified Stöber	DNA embedded into silica nanoparticles	Biocompatible, simple, scalable, transfection	Limited to DNA loading, reduced uptake with larger particles	Delivery of plasmids and DNA; efficient transfection; low cytotoxicity; gene expression studies	[26]
	Stöber-like with templating agent (CTAB, PEI)	DNA loaded in MSNs	High transfection efficiency	Efficiency varies with cell redox state	Delivery of DNA, siRNA, anti-miRNA, mRNA; endosomal escape; cancer therapy; hard-to-transfect cells	[66]
	Stöber with Head–Tail Growth	Asymmetric silica nanoparticles modified with PEI, DNA-loaded	Controlled DNA release, improved uptake, low cytotoxicity	Tail length affects DNA binding	Efficient plasmid DNA delivery; superior to spherical/dendritic MSNs	[67]
	CTAB-assisted Stöber + lipid coating	Positively charged MSNs functionalized with APTES, nucleic acids complexed, lipid coated	High gene activation, endosomal escape, CRISPR/Cas protection, targeted delivery	Complex design, limited scalability	DNA/plasmid delivery; bladder cancer gene therapy; targeted nucleic acid delivery	[38]
	CTAB-directed Stöber (Drug + Gene)	DOX-loaded COOH-MSNs with siRNA chimeras	Controlled DOX release, targeted uptake, reduced toxicity	Complex, multistep nucleic acid encapsulation requiring polymer and chimera modification	Co-delivery of siRNA and anticancer drugs; resistant bladder cancer therapy	[68]
	CTAB-directed Stöber (nDNA complexes)	nDNA + MSNs at various N/P ratios	Effective internalization, miR-21 knockdown	Encapsulation depends on PEI and precise nucleotide modification	miRNA delivery; tumor growth reduction; surface modifications improve efficiency	[69]
	CTAB-directed Stöber	Functionalized MSNs with APTES, siRNA loaded	Up to 98% silencing efficiency in plants	Possible leaf damage	Transient gene knockdown in plants; exogenous/endogenous gene silencing	[70]
	CTAC-modified Stöber	Ultrasmall MSNs with PEI, anti-miRNAs loaded	Reduced tumor spheroid size, sustained release, DNA protection	Excess PEI hinders release	miRNA delivery in brain cancer cells; efficient uptake	[43]
Sol-gel and hybrid organosilica	Sol-gel procedure	mRNA premixed with cationic polymer, bound to MSN surface	Improved *in vitro/in vivo* transfection; stable; scalable	Synthesis affects expression/stability	Targeted siRNA and anticancer drug delivery	[49]
	Sol-gel + CTAB (DMONs-ZIF-8)	mRNA in PEI-coated DMONs-ZIF-8	High loading, enhanced transfection, biocompatibility, superior to PEI and lipofectamine	None reported; highly efficient	Efficient mRNA delivery with effective endosomal escape, RNA protection, and strong *in vitro/in vivo* transfection	[71]
	Sol-gel + CTAB (vaccine)	mRNA in PEI-coated MSNs	Local and prolonged protein expression, robust immune response	Optimization needed for nucleotide modifications	Vaccine development; T-cell response; neutralizing antibodies	[72]
Bottom-up/stimuli-responsive/spiky	Bottom-up/stimuli-responsive	DNA integrated within silica scaffolds	Stimuli-responsive release, uptake in cancer cells	Complex design	Controlled release of DNA/RNA sensitive to temperature, ATP, etc.; drug/gene delivery; biosensing	[73]
	Spiky-assisted silica growth	CaClOH-modified spiky silica nanoparticles	Endosomal escape, sustained Ca^2+^ release, enhanced mRNA translation	Reduced expression with RNAse; higher PEI cytotoxicity	mRNA delivery *in vivo*; enhanced translation via mTORC1 activation	[74]
	Basic catalysis + lipid bilayer	RNPs of CRISPR/Cas loaded in LCMSNs	Efficient infection biocompatible; versatile	Complex design; limited loading	Delivery of CRISPR/Cas9 RNPs for host gene editing and viral prevention	[36]
Reverse microemulsionn/Biphasic/Top-Down	Modified reverse microemulsion	DNA encapsulated in ion-doped hollow silica nanoparticles	High ion incorporation, controlled release, DNA protection, scalable	Uptake depends on ion type, DNA concentration, HSN dose	Oligonucleotide and DNA delivery; protection from degradation; one-pot ion/dye co-doped nanoparticles	[75]
	Oil–Water biphasic stratification (CTAB)	Two-stage synthesis of amine-functionalized, dye-labeled MSNs	Optimal size, structural control, reduced aggregation	Unsuitable for brain delivery; need enzymatic protection	mRNA encapsulation; ADP-MSNs with narrow size distribution; functional group control	[76]
	Top-down nanozeolitic + CTAB	ZSM-5 template dissolved, modified with APTMOS	High mRNA/pDNA encapsulation, nuclease protection	pDNA encapsulation did not enhance immunogenicity	mRNA encapsulation enhances humoral immune response	[77]
Scaffold/Electrosp.	TEOS-NH_3_ + L-arginine-HCl + CTAB/HTAB	DNA adsorbed to silica nanoparticles in electrospun scaffolds	Low cytotoxicity, controlled release, DNA protection	DNA re-adsorption; moderate cytotoxicity with PEI	Tissue engineering; cell-based therapy; slow DNA release for transfection	[78]
	Electrospraying of pre-synthesized MSNs	siRNA loaded into PEI/phospholipid-coated MSNs	High encapsulation, effective siRNA delivery	High *in vitro* cytotoxicity; needs animal validation	siRNA delivery; reduced chemotherapy doses	[65]

**Abbreviatures: TEOS**: tetraethyl orthosilicate; **TEA**: Triethanolamine; **CTAB**: cetyltrimethylammonium bromide; **FC4**: Sodium heptafluorobutyrate; **TBOS**: tetrabutyl orthosilicate; **BTES**: bis(triethoxysilyl)propane tetrasulfide; **PEI**: polyethyleneimine; **MONs**: Mesoporous organosilica nanoparticles; **MSNs**: inorganic mesoporous silica nanoparticles; **RITC**: Rhodamine B isothiocyanate; **APTES**: (3-Aminopropyl) triethoxysilane; **DSPE-PEG**: distearoylphosphatidylethanolamine-hydrazone-polyethylene glycol; **PLZ4**: cyclid peptide ligand specific to bladder cancer cells; **ASOs**: antisense oligonucleotides; **APTMOS**: 3-(Aminopropyl) trimethoxysilane **HAS**: Hierarchical Aluminosilicate; **DOX**: Doxorrubicine; **DMONs**: Dendritic Mesoporous Organosilica Nanoparticles; **ZIF-8**: Zeolitic Imidazolate Framework-8; **LCMSNs**: lipid coated mesoporous nanoparticles; **RNPs**: ribonucleoproteins.

#### 2.1.2 Surface coating for stealth and identity

Once the internal architecture of MSNs is optimized, the outer surface becomes the dominant factor shaping biological interactions *in vivo*. Bare silica rapidly adsorbs form a dynamic protein corona that alters ζ-potential, hydrodynamic size, and overall “biological identity”, typically accelerating opsonization and clearance by the mononuclear phagocyte system [35,79]. Although corona formation is inevitable, it can be modulated through rational surface engineering. Indeed, surface functionalization is widely regarded as the most critical design parameter governing the *in vivo* fate of MSNs. Poly(ethylene glycol) (PEG) remains a classical strategy to achieve colloidal stability and stealth behavior, creating a hydrated steric barrier that suppresses protein adsorption and mononuclear phagocyte system (MPS) uptake. However, repeated administration can induce anti-PEG antibodies and complement activation, compromising long-term stealth. Recent studies have demonstrated the importance of brush conformation and grafting density in modulating this trade-off, prompting interest in mixed or cleavable PEG architectures [80,81].

Zwitterionic coatings, based on sulfobetaine or phosphorylcholine, have emerged as robust alternatives to PEG for silica systems, forming ultra-hydrated shells that resist nonspecific adsorption in complex media. Sulfobetaine-modified MSNs exhibit strong antifouling behavior and colloidal stability under physiological ionic strength [82,83]. Complementarily, pseudo-zwitterionic MSNs, incorporating amino and phosphonate groups, can further reduce corona formation and macrophage uptake compared to PEG-MSNs [84]. Moreover, phosphorylcholine (MPC) coatings have been grafted onto biodegradable MSNs to reinforce low-fouling, immunologically neutral interfaces [85].

Beyond synthetic coatings, bioinspired surface functionalizations are redefining how silica nanoparticles interact with biological systems. The integration of natural cell membranes or extracellular vesicles (EVs) onto MSNs creates biomimetic surfaces capable of immune evasion, self-recognition, and selective accumulation in disease-relevant tissues. EV-coated MSNs can retain the targeting properties of their parental cells, enabling selective internalization and enhanced delivery of cytotoxic payload to tumor cells [86]. Similarly, osteoblastic cell membranes coatings confer homotypic tropism toward bone microenvironments, suggesting that the biological origin of the coating governs biodistribution and tissue affinity [87]. Expanding this paradigm, immune-cell-based cloaks, such as natural killer (NK) membrane-cloaked disulfide-bridged organosilica “nanogenerators”, have enable prolonged circulation, tumor homing, and activatable behavior under near-infrared (NIR) irradiation [88].

Table 2 presents a comparative summary of surface functionalization strategies applied to silica nanoparticles and their biological consequences. This overview captures how different coatings, from synthetic polymers to biomimetic membranes, modulate key parameters such as colloidal stability, protein corona formation, immune evasion, and targeting specificity. By mapping these effects to specific chemistries, the table highlights the pivotal role of surface design in shaping the *in vivo* behavior and therapeutic potential of silica-based gene delivery systems.

**Table 2:** Surface functionalization strategies and biological effects of silica nanoparticles.

Coating type	Primary purpose	Effect on biodistribution	Immunological risk	Applications	Ref.
PEG	Colloidal stability/stealth	Prolonged circulation	Anti-PEG response	siRNA in solid tumors	[89,90]
Zwitterionic	Antifouling/stealth	Low RES uptake	Low	mRNA in sensitive tissues	[91,92]
Cell membrane	Biomimetic/targeting	Homotypic tropism	Very low	CRISPR/Cas in brain/bone	[[93], [94], [95]]
Aptamers	Specific recognition	Directed accumulation	Low	AS1411/TfR targeting	[96]
Antibodies/fragments	High specificity	Tumor-selective uptake	Moderate	HER2/EGFR targeting	[97]
Electrostatic ligand Protein coating	Improved tageting	Accumulation in tumors	Moderate	GB3; VEGFr; TEM8 in soid tumors	[25]

### 2.2 Precision targeting strategies

Rational ligand display on MSNs converts passive biodistribution into receptor-directed accumulation and cellular uptake, reducing off-target exposure and improving intracellular delivery of nucleic acids. High density of surface silanol on silica enables robust covalent conjugation of ligands while preserving core integrity. Crucially, ligand orientation, spacer length, and grafting density govern avidity and accessibility in protein-rich media and must be co-optimized with the stealth layer [98].

Peptide ligands, particularly the integrin-binding Arg-Gly-Asp (RGD) peptide, facilitate adhesion and internalization primarily in tumor cells through overexpressed integrin receptors, with more limited or context-dependent interactions with vascular endothelial cells. When couples with stimulus-responsive gatekeepers, RGD-functionalized MSNs enable selective recognition and triggered release in endosomal or mildly acidic conditions. Temperature- and pH-responsive RGD-MSN systems exemplify this synergy between targeting and controlled payload exposure [99]. Similarly, cell-penetrating and localization peptides, such as the trans-activator of transcription (TAT) peptide and nuclear localization signals (NLS), have been employed to enhance cellular uptake or direct subcellular trafficking. Their efficacy depends on linker design and spatial presentation above the stealth brush to avoid marking by the protein corona [98]. Aptamers provide compact, non-immunogenic recognition, including transferrin receptor (TfR)-binding aptamers or AS1411 targeting nucleolin, and can be combined with PEGylation or magnetic guidance; dual-targeting strategies (e.g., AS1411 combined with hyaluronic acid/CD44 or RGD) further improve selectivity in heterogeneous tissues [100]. Antibody or antibody-fragment targeting of epidermal growth factor receptor (EGFR) or human epidermal growth factor receptor 2 (HER2) is compatible with responsive shells or hybrid coatings and can improve uptake in antigen-overexpressing tumors [101].

Across peptides, aptamers, and antibodies, three cross-cutting design principles consistently emerge for MSN-based targeting: (i) decouple stealth and targeting by using a low-fouling base layer (PEG or zwitterionic) and projecting ligands beyond them with optimized spacer; (ii) tune ligand surface density to balance avidity and mobility, avoiding corona-induced masking; (iii) implement dual-receptor or hierarchical targeting to address tissue heterogeneity and enhance intracellular delivery. These principles are recurrently emphasized in recent silica-focused reviews and experimental studies on siRNA and mRNA carriers [102].

### 2.3 Safety and biodistribution

After systemic administration, MSNs primarily accumulate in liver and spleen. Their biodistribution is governed by particles size, aspect ratio, surface charge, and interfacial chemistry. Notably, shape effects are pronounced: short-rod MSNs enrich in liver, whereas long-rod variants tend to enrich in the spleen [35,103]. Particles smaller than ∼10 nm are rapidly renally filtered, while larger carriers require biodegradation into soluble species prior to elimination. Interestingly, atypical clearance pathways have been reported when surface chemistry facilitates translocation across the glomerular barrier [35,104]. Silica is predominantly cleared via hydrolytic dissolution of Si–O–Si bonds to orthosilicic acid (Si(OH)_4_), a physiological metabolite excreted in urine. Dissolution rate depends on framework condensation, porosity, organic bridging (e.g., disulfide/tetrasulfide in periodic mesoporous organosilica), allowing scaffolds persistence to be tuned from hours to weeks [[105], [106], [107]]. Incorporating degradable linkers accelerates breakdown in reductive intracellular conditions, such as elevated glutathione (GSH) levels, reducing long-term reticuloendothelial system (RES) retention without sacrificing loading capacity [106,107].

Complete removal of surfactant is critical: residual template correlates with hemolysis and hepatic stress, whereas template-free MSNs exhibit improved tolerability. Acidic ethanol washes, dialysis, or calcination are effective and substantially improve biocompatibility [[108], [109], [110]]. Across recent *in vivo* studies, well-engineered MSNs (≈50–200 nm) with low-fouling surfaces show minimal chronic toxicity at therapeutic doses, transient macrophage activation, and progressive silicon clearance as dissolution proceeds [35,55].

In summary, the systemic fate of silica nanoparticles is dictated by a delicate interplay between scaffold architecture, surface chemistry, and biodegradability. Evidence from recent *in vivo* studies consistently highlights that rationally engineered MSNs can achieve safe circulation, predictable clearance, and minimal long-term toxicity when degradable linkers, low-fouling coatings, and harmonized pharmacokinetic endpoints are incorporated [105]. These insights establish a robust foundation for addressing the next critical challenge: overcoming intracellular barriers such as endosomal escape, cytoplasmic release, and nuclear trafficking, which ultimately determine the therapeutic efficacy of nucleic acid delivery.

**Fig. 2:**
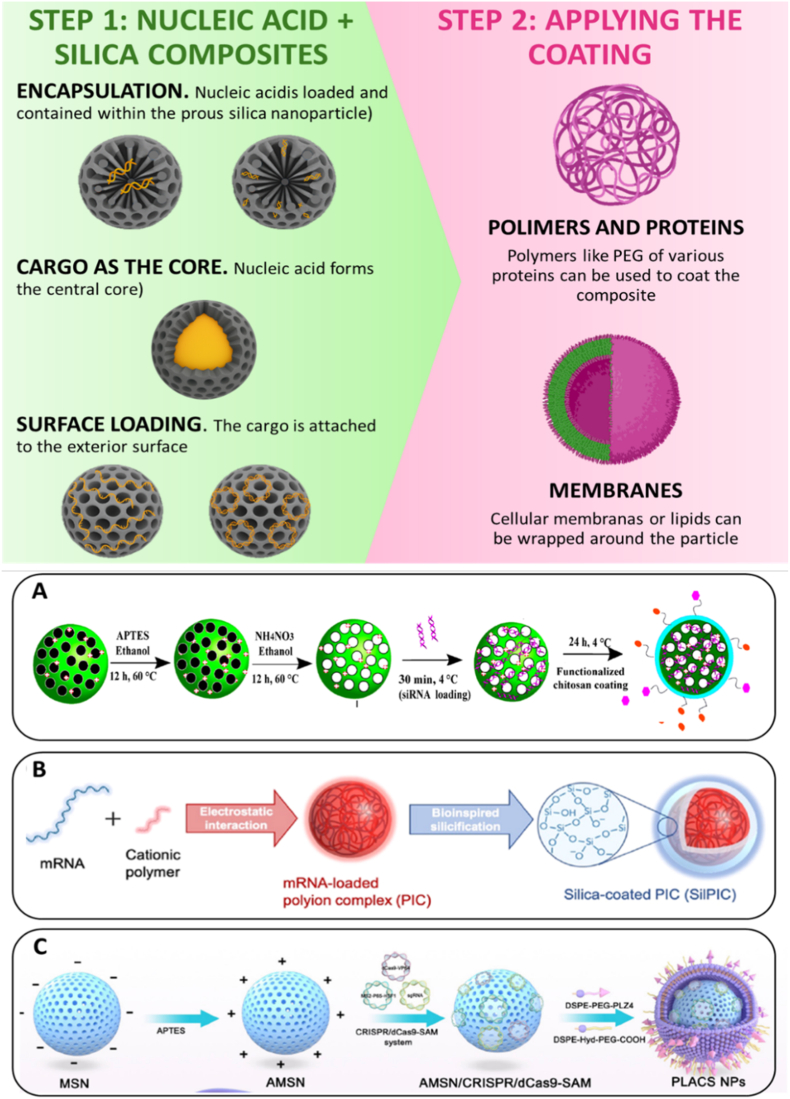
**Engineering strategies for silica–nucleic acid composite formation. Top panel:** Overview of the two-step design process. Step 1 illustrates nucleic acid integration into silica nanostructures via (i) encapsulation within mesopores, (ii) core incorporation, or (iii) surface loading. Step 2 shows subsequent application of protective or functional coatings using polymers/proteins or lipid/cell membrane wrapping to enhance stability, immune evasion and targeting. **Bottom panel**: Representative examples of these strategies from the literature. (A) Example of encapsulation of a small nucleic acid (siRNA) into mesoporous silica nanoparticles, followed by chitosan-based PEG–folate and PEG–TAT coating. Image modified from Heidari et al. [45]. (B) Example of cargo-as-core strategy, where an mRNA-loaded polyion complex is formed via electrostatic interaction and subsequently silica-coated [47]. *Copyright © 2021, American Chemical Society*. (C) Example of surface loading and PEG-based coating, where CRISPR/dCas9-SAM complexes are adsorbed onto APTES-modified MSNs and further protected with PEG-based lipidic layers [38]. *Copyright © 2025 Elsevier B.V.*

The main strategies used to assemble silica–nucleic acid composites and apply interfacial coatings, which directly influence intracellular delivery performance, are schematically illustrated in Fig. 2.

## 3 Engineering the cargo release: overcoming intracellular barriers

### 3.1 Strategies for endosomal escape

**Fig. 3:**
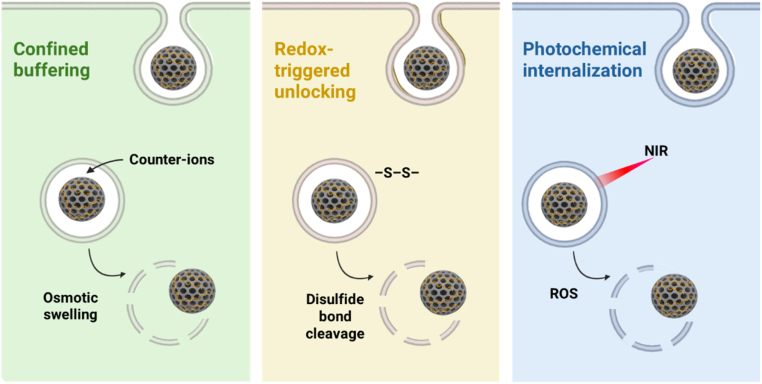
**Schematic illustration of representative endosomal escape strategies for mesoporous silica nanoparticles.** In green, the confined buffering or proton-sponge effect shows weakly basic surface groups (amines, imidazole) that buffer endosomal acidification, leading to counter-ions and H_2_O influx, osmotic swelling, and membrane rupture. In yellow, the redox-triggered unlocking mechanism illustrates glutathione (GSH)-responsive disulfide (–S–S–) linkages that are cleaved under the reductive cytosolic environment, opening the mesopores and releasing the cargo. In blue, the photochemical internalization strategy depicts near-infrared irradiation activating photosensitizers on the MSN surface, generating reactive oxygen species (ROS) that oxidatively disrupt the endosomal membrane and promote cytosolic delivery.

Following endocytosis, MSNs must rapidly breach the endosomal membrane to prevent lysosomal degradation and enable cytosolic delivery of the therapeutic payload. Three complementary design strategies dominate: (i) confined buffering, often referred to as the “proton-sponge” effect, achieved by integrated weakly basic groups or near the mesopores to induce osmotic swelling and subsequent rupture driven by the buffering capacity of polycations, thereby releasing the cargo into the cytoplasm [111]; (ii) environment-cleavable architectures, primarily based on GSH-responsive disulfide linkages, which unlock pores or disassemble interfacial linkers under reductive intracellular conditions; (iii) Photochemical internalization (PCI) employs light-activated photosensitizers that accumulate on endosomal membranes and, upon irradiation, generate reactive oxygen species (ROS) to disrupt the membrane and release the cargo into the cytosol [112,113]. Across all three strategies, the spatial localization of active moieties, whether within the pore interior, at the pore mouth, or embedded in a thin shell, is as critical as their chemical identity. Misplacement or excessive external cationic charge can promote protein corona formation, hinder endosomal escape, or increase cytotoxicity [114]. These strategies are schematically summarized in Fig. 3.

**Fig. 4:**
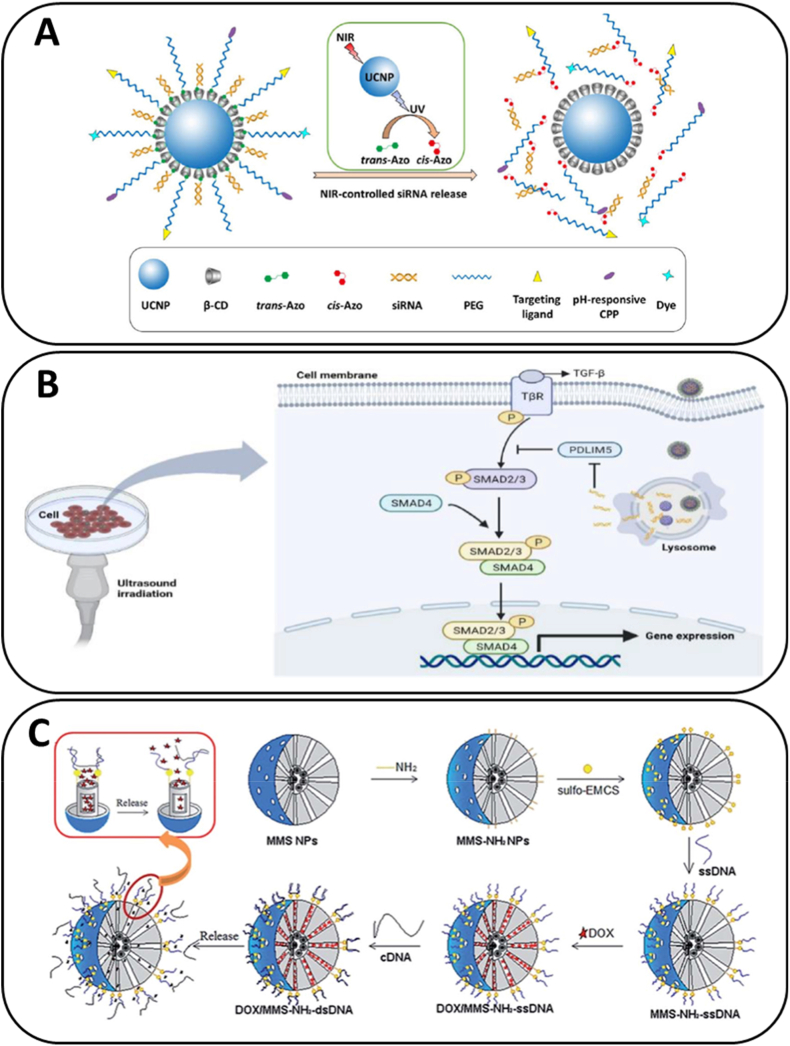
**Representative examples of stimuli-responsive release strategies using silica-based nanocarriers for nucleic acid delivery.** (A) **Near-infrared (NIR) activation:** silica-based platforms incorporating azobenzene gatekeepers and upconversion nanoparticles enable NIR-triggered release of siRNA [115]. *Copyright© 2017 Elsevier B.V.* (B) **Ultrasound-triggered delivery:** mesoporous silica nanoparticles facilitate ultrasound-enhanced release and intracellular silencing of siRNA [116]. *Copyright © 2025 Elsevier B.V* (C) **Temperature- and magnetically responsive system:** Fe_3_O_4_/SiO_2_ magnetic mesoporous silica nanoparticles capped with DNA enable temperature-controlled release and support magnetically induced hyperthermia for nucleic acid or drug delivery [117]. *© The Royal Society of Chemistry 2015*.

#### 3.1.1 Confined buffering

Weakly basic functionalities such as aminosilanes, PEI segments, and imidazole/histidine, motifs buffer endosomal acidification, recruit counter-ions and water, and generate the osmotic pressure required stress needed for vesicle destabilization (see Fig. 4). Localizing these groups within mesopores or at pore mouths focusses buffering where it is most needed, while maintaining a moderate external ζ-potential. This configuration supports nucleic acid complexation and cytosolic transfer with reduced hemolysis risk compared to thick polycationic outer coatings. Kinetic analysis using MSN–siRNA system show that vectors must actively promote endosomal escape to achieve gene silencing. Surface composition and buffering capacity were decisive for cytosolic access [114]. Histidine-functionalized MSNs can outperform amine-only controls for plasmid DNA delivery (consistent with buffering near pH ≈ 6.0) [118].

Silica carriers that combine adequate pore entrance dimensions (≥∼4–5 nm) with multifunctional polymer caps further enhance siRNA loading and endosomal release while mitigating polycation toxicity through architectural precision rather than excessive charge density [118]. Recent silica-focused reviews consistently emphasize a key design principle: confine buffering moieties and minimize external cationic exposure to balance escape efficiency and biocompatibility [119].

#### 3.1.2 Redox-triggered unlocking

Endosomal escape and cytosolic release can be strategically coupled to intracellular GSH levels by installing disulfide (–S–S–) linkages into the silica scaffold, either at the pore entrance (as caps or gatekeepers) or within a thin organosilica interface. In MSNs loaded with siRNA, the disulfide bonds function as “molecular fuses”: following endosomal trafficking, the reductive cytosol environment cleaves the S–S bond, triggering pore opening and facilitating the release of the nucleic-acid cargo. This process is often accompanied by partial interfacial softening, which further promotes membrane destabilization and escape. Representative MSNs system shows co-delivery of siRNA and doxorubicin in which disulfide-linked siRNA served as a gatekeeper. Upon GSH-mediated cleavage, both siRNA and Dox were released in a synchronized manner, resulting in enhanced gene knockdown efficacy *in vitro* and *in vivo* [120]. Silica-focused reviews emphasize positioning cleavable elements at accessible sites, typically at the pore mouth or within a degradable shell, where they remain exposed and functional despite protein corona formation [121].

#### 3.1.3 Photochemical internalization

Photochemical internalization (PCI) couples spatiotemporally controlled light irradiation with photosensitizer-mediated endosomal rupture. After cellular uptake, photosensitizers preferentially accumulate in endosomal and lysosomal membranes. Upon illumination, they generate short-lived reactive oxygen species (ROS) that oxidatively disrupt the lipid bilayer, producing transient membrane permeabilization and releasing the encapsulated nucleic acids or drugs into the cytosol. Unlike buffering- or redox-based strategies, which rely on intrinsic physicochemical cues, PCI is externally activatable and can be applied on demand to specific regions, thereby limiting off-target cytotoxicity.

A representative implementation uses upconversion nanoparticles (UCNPs) coated with a mesoporous silica shell to co-load (i) a photosensitizer (e.g. hypocrellin A) and (ii) a therapeutic nucleic acid payload such as siRNA. The UCNP core converts tissue-penetrant near-infrared (NIR) light (≈980 nm) into shorter-wavelength emission that activates the photosensitizer locally. The resulting ROS production weakens the endosomal membrane and promotes efficient endosomal escape of the siRNA, which in turn improves target gene silencing and suppresses tumor growth *in vitro* and *in vivo*. This modular “photo-tearable” MSN–UCNP architecture highlights two advantages of PCI in the MSN context: (i) on-demand release controlled by an external trigger (light), and (ii) enhanced cytosolic delivery without requiring high external cationic charge density.

### 3.2 Stimuli-responsive release

External stimuli (light, ultrasound, alternating magnetic fields) can be strategically integrated into mesoporous silica scaffolds to enable controlled release of nucleic acids with spatial and temporal precision. These triggers can unlock pore gates, facilitate endosomal escape, or accelerate cytosolic release. A consistent design principle across these systems is the placement of the transducer element (photothermal, photochemical, acoustic, or magnetic) at the core–shell interface or pore entrance, allowing the applied stimulus to modulate local permeability or cargo binding without compromising colloidal stability [121].

#### 3.2.1 Light triggers (NIR/visible)

Light-responsive silica platforms have been extensively explored using two main approaches: plasmonic heating and upconversion photochemistry. Silica–gold nanoshells (SiO_2_@Au) convert NIR to local heat, weakening electrostatic interactions or melting duplex caps for on-demand siRNA release. Mechanistic studies have shown that laser fluence and pulse duration govern the transition between photothermal release and photoporation, offering precise spatiotemporal control over gene silencing [122,123]. Alternatively, silica-coated upconversion nanoparticles (Si-UCNPs) absorb tissue-penetrating NIR light and emit UV or visible photons locally. These emissions can cleave photocaged linkers or activate photoisomerizable caps, such as azobenzene/β-cyclodextrin complexes, installed at the pore entrance. This strategy enables NIR-triggered siRNA release while maintaining low surface charge, with proof-of-concept studies demonstrating effective gene knockdown *in vitro* and *in vivo* [115,124].

Visible-light-responsive molecular nanogates, including azobenzene/β-cyclodextrin and nanopiston-like designs, provide changes from “closed” to “open” allowing controlled nucleic acid release [125]. For optimal performance, gate chemistries and photocages should be positioned at the pore entrance, while NIR-responsive transducers must be placed in close proximity to maximize release efficiency, minimize nonspecific heating, and preserve nanoparticle integrity [122,124].

#### 3.2.2 Ultrasound triggers

Ultrasound (US) offers a clinically mature and noninvasive stimulus capable of penetrating deep tissues. In MSN-based platforms, US can be harnessed to disrupt supramolecular caps, enhance endosomal escape through transient membrane permeabilization (sonoporation), or actuate microbubble-assisted delivery, where silica serves as the reservoir for drugs or siRNA.

Several studies have demonstrated that US-responsive MSNs, as well as lipid- or microbubble-assisted silica constructs, can achieve efficient siRNA binding and transfection. These systems exhibit improved intracellular release when exposed to diagnostic-range ultrasound. Notably, hybrid magnetic MSNs encapsulated within microbubbles have been specifically engineered for ultrasound-mediated imaging and gene transfection, highlighting a translatable strategy for externally triggered nucleic acid delivery [63,126].

Recent advances have further validated the practicality of ultrasound-mediated gene regulation using silica carriers. For instance, MSN systems loaded with disease-relevant siRNAs, such as those targeting PDZ and LIM domain protein 5 (PDLIM5), have shown efficient complexation and release upon US, supporting their potential for on-demand therapeutic modulation [116].

#### 3.2.3 Magnetic and thermally triggered release

Embedding magnetic nanodomains within mesoporous silica architectures, either as core@shell or rattle-type magnetic MSNs (MMSNs), enables actuation via alternating magnetic fields (AMF). Localized magnetic heating induced by AMF can accelerate gate opening or promote thermal dehybridization of nucleic acid caps, allowing remote control of cargo release without the need for optical stimuli. Silica-encapsulated magnetic platforms have demonstrated AMF-triggered release profiles within thermal windows compatible with cell viability. The SiO_2_ shell not only provides a porous matrix for oligonucleotide loading but also stabilizes the magnetic core, reducing aggregation and minimizing cytotoxicity. A representative example includes DNA-capped Fe_3_O_4_/SiO_2_ mesoporous nanocarriers, which exhibit temperature-controlled gate opening and efficient magnetic heating, serving as a reliable switch for nucleic acid release [117].

To consolidate the diverse design strategies and functional outcomes described above, Table 3 summarizes representative silica-based platforms engineered for stimulus-responsive nucleic acid release. Each entry highlights the nature of the external trigger, its mechanism of action, tissue penetration depth, and typical genetic cargo, along with illustrative examples and key references. This comparative overview underscores the versatility of mesoporous silica nanoparticles as transducer-integrated carriers capable of precise, externally guided gene delivery.

**Table 3:** External stimuli for controlled nucleic acid release from silica nanoparticles.

External Stimulus	Mechanism of Action	Penetration Depth	Typical Application	Example Platform	Ref.
NIR Light	Photothermal/photochemical	Moderate–High	siRNA, mRNA	SiO_2_@Au, Si-UCNPs, UCNP@SiO_2_	[124,127]
Visible Light	Isomerization/bond cleavage	Low	siRNA	Azobenzene nanogates, β-cyclodextrin	[128]
Ultrasound	Sonoporation/microbubble disruption	High	siRNA, CRISPR/Cas	Microbubble–MSNs	[63]
Magnetic Field/Temperature	Local hyperthermia/dehybridization	High	DNA, siRNA	Fe_3_O_4_@SiO_2_ MMSNs,	[64]
pH/Redox	Bond cleavage/scaffold dissolution	Intracellular	mRNA, siRNA	Disulfide-bridged MONs, Disulfide-gated MSNs	[71,120,129]

## 4 The silica platform for advanced gene expression manipulation

Delivering macromolecular genetic payloads, particularly mRNA and CRISPR/Cas ribonucleoproteins (RNPs), requires carriers that provide high loading capacity, protect fragile cargo during processing/circulation, and enable on-demand release. MSNs and related organosilica/hollow architectures address these limitations via (i) high internal surface area, (ii) tunable pore architecture (typically ≈2–50 nm), and (iii) robust inorganic frameworks that protect fragile cargos during processing/circulation while enabling programmable release. Early structure–function studies established that enlarging pore apertures and controlling topology are critical for accommodating bulky biomacromolecules without denaturation—principles now leveraged for nucleic acid therapeutics [130]. These same architectural features also suggest that silica-based platforms may be well suited to accommodate emerging RNA modalities, such as circular RNA (circRNA) and self-amplifying RNA (saRNA), which impose increased demands on cargo size and stability [131,132].

### 4.1 Large cargo delivery

From a design perspective, the central challenge is to accommodate large, labile constructs such as long mRNA and CRISPR/Cas RNPs while preserving biological activity and achieving cytosolic availability. In practice, successful silica platforms converge on a common set of strategies. First, pore entrance and internal geometry are matched to the hydrodynamic dimensions and flexibility of the payload to ensure high-capacity loading and unhindered diffusion, with large-pore and hierarchical MSNs proving especially effective [130]. Second, “chemistry-in-framework” approaches incorporate cleavable motifs, tetrasulfide linkers are a prominent example, that render the matrix degradable under intracellular redox conditions, aligning disassembly and release kinetics with the cytosolic environment and outperforming non-degradable analogues for nucleic-acid transfection [66]. Third, interfacial complexation is exploited to stabilize polyanionic cargo without sacrificing the mechanical advantages of the silica scaffold; polyplex-on-silica architectures exemplify how electrostatic bridging can protect RNA during trafficking while maintaining a well-defined particle morphology [49]. Finally, transmembrane transport and endosomal escape are enhanced by adding fusogenic or lipidic layers to the silica surface, thereby combining the volumetric and mechanical benefits of an inorganic core with the membrane interaction profile of soft matter. Lipid-coated MSNs (LC-MSNs) are the most mature embodiment of this concept [52]. Architectural variants such as hollow silica nanoparticles (HSNs) further extend volumetric capacity by providing a central void for encapsulation while preserving an outer shell for targeting and stealth, thus increasing payload density without compromising colloidal or mechanical stability [133]. Table 4 cross-references these structural levers with the biophysical requirements of distinct genetic modalities, providing a concise map from molecular size and degradation sensitivity to pore compatibility, trigger chemistry and representative particle designs.

**Table 4:** Comparison of genetic cargo types and structural requirements.

Genetic Cargo Type	Approximate Size	Pore Size Requirement	Degradation Sensitivity	Release Requirement	Example Platform	Ref
siRNA	∼13 kDa	2–5 nm	Moderate	Endosomal escape	PEI-coated MSNs	[102,114,134,135]
mRNA	∼300–500 kDa	≥10 nm	High	Redox-responsive	Tetrasulfide MONs	[66,71]
Plasmid DNA	>1 MDa	≥15 nm	High	Sustained release	Hollow MSNs	[119,133]
CRISPR/Cas9 RNP	∼160 kDa	≥10 nm	High	Endosomal escape + stealth	LC-MSNs	[36,52]

#### 4.1.1 mRNA delivery

mRNA delivery benefits from an interplay between architectural optimization for capacity and framework chemistry for stimulus-responsive release. A recent *in vivo* study systematically varied particle size, porosity, surface topology and aspect ratio to delineate how each parameter shapes adsorption, nuclease protection and translational output. The resulting polyplex-on-silica formulation, prepared by premixing mRNA with a cationic polymer and binding the complex to the silica surface, achieved efficient expression *in vivo*, thereby illustrating how silica's mechanical robustness can be functionally decoupled from interfacial design to tune pharmacological performance [49] In parallel, mesoporous organosilica nanoparticles bearing tetrasulfide bridges introduce redox-triggered degradability while preserving circulation stability; these materials consistently enhance nucleic-acid transfection relative to non-degradable counterparts by synchronizing framework disassembly with the intracellular reducing milieu [66]. Together, these lines of evidence support a dual design logic for mRNA: pore-scale engineering to maximize loading and diffusion of long transcripts, and chemistry-in-framework to orchestrate cytosolic release with minimal loss of integrity.

#### 4.1.2 CRISPR/Cas RNP delivery

**Fig. 5:**
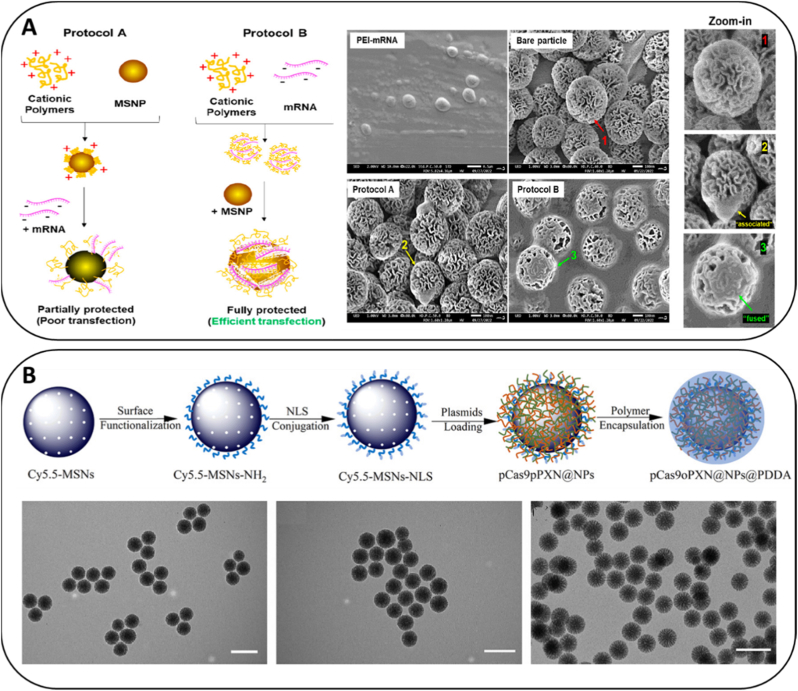
**Comparative encapsulation strategies for mRNA and CRISPR/Cas9 plasmids using mesoporous silica nanoparticles.** (A) Schematic illustration of preparing the MSNs and mRNA/PEI-MSNs mixture using protocol A or protocol B and SEM images of PEI-mRNA, the bare particle, and the mRNA/PEI-MSNP mixture using protocol A and protocol B [49]. *Copyright © 2023 American Chemical Society*. (B) Schematic illustration of the functionalized MSNs-based nanocarrier for the delivery of CRISPR/Cas9 plasmids: preparation of Cy5.5-MSNs-NLS, plasmid loading, and polymer encapsulation and TEM micrographs of all stages. Scale bar: 200 nm [136]. *Copyright © 1999-2025 John Wiley & Sons*.

The dimensional and compositional complexity of CRISPR/Cas RNPs, on the order of ∼160 kDa protein plus guide RNA, demands substantial free volume and a microenvironment that preserves conformational integrity while enabling timely cytosolic access. Silica-based carriers meet these requirements through hybrid constructs that marry an inorganic core with membrane-active interfaces. Functionalized mesoporous silica nanoparticles have also been shown to support efficient CRISPR/Cas9-mediated genome editing, enabling green fluorescent protein (GFP)-tagged paxillin knock-in via delivery of plasmid-based CRISPR/Cas9 systems and demonstrating that appropriately engineered MSNs can sustain intracellular trafficking and nuclear access of large gene-editing constructs [136] (Fig. 5B). LC-MSNs use a mesoporous silica core to provide encapsulation capacity and mechanical stability, while a biomimetic lipid envelope promotes cellular uptake, membrane fusion and endosomal escape. Foundational studies demonstrated delivery of CRISPR/Cas RNPs and plasmids with efficient *in vivo* editing, establishing LC-MSNs as a credible chassis for genome engineering [52]. Building on this platform, subsequent work delivering CRISPR/Cas RNPs against NPC1 reported reduced viral infection, highlighting the translational promise of silica-based vehicles for antiviral genome editing [36]. Complementary reports have shown efficient CRISPR/Cas editing with low off-target activity using silica systems in oncology settings [119], while calcium-modified silicas have improved uptake and translation of RNA cargos without compromising structural integrity [74]. Collectively, these advances position silica architectures as particularly well suited for “large cargo” genome engineering, where volumetric accommodation, controlled disassembly and effective endosomal escape must be satisfied in concert.

### 4.2 Combinatorial and theranostic modularity

The modular architecture of MSNs provides a versatile foundation for integrating multiple therapeutic and diagnostic functions within a single platform. Across the wide range of combinatorial systems reported to date, three recurring design principles guide their rational engineering. Biological complementarity underpins the selection of cargos that modulate convergent or synergistic pathways to enhance efficacy, overcome drug resistance, or reshape the tumour microenvironment [[137], [138], [139]]. Physicochemical compatibility exploits the tunable pore structure, surface chemistry and compartmentalization of MSNs to co-encapsulate cargos with distinct sizes, charges and hydrophobicities [71,140]. Spatiotemporal control, achieved through stimuli-responsive gates or hierarchical loading schemes, enables coordinated release in response to pH, redox or external triggers [137,141,142]. These principles collectively frame the major MSN-based combination strategies discussed below.

#### 4.2.1 Co-delivery of nucleic acids and small-molecule drugs

Co-delivery of nucleic acids and small-molecule drugs enables synergistic antitumour effects by combining pathway modulation with cytotoxicity and is particularly valuable in chemoresistant tumors, where siRNA or miRNA can sensitize cells to chemotherapy by downregulating efflux pumps or survival pathways [119]. MSNs are especially well suited for this approach owing to their large pore volume and versatile surface functionalization, which allow independent optimization of loading efficiencies for hydrophobic drugs and nucleic acids while preserving nucleic acid integrity [119,143]. At the same time, the physicochemical disparity between cargos introduces challenges related to harmonizing release kinetics, preventing premature drug leakage and maintaining nucleic acid stability during intracellular trafficking [4,139].

**Fig. 6:**
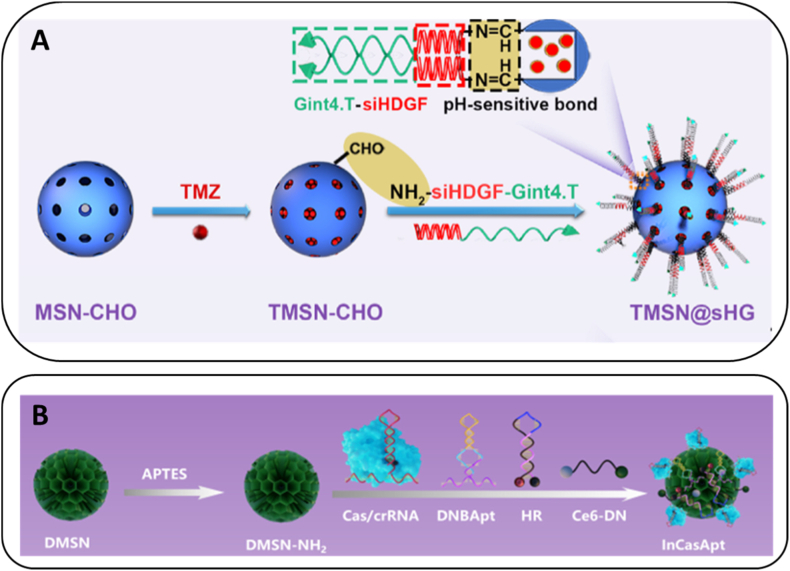
**Representative examples of co-delivery strategies using mesoporous silica nanoparticles.** (A) Drug + nucleic acid co-delivery: Schematic illustration of pH-responsive mesoporous silica nanoparticles (TMSN@siHG), prepared by loading temozolomide (TMZ) into aldehyde-functionalized MSNs (MSN-CHO) and subsequently capping the surface with a Gint4.T–siHDGF chimera via acid-labile benzoic–imine linkages. The resulting nanosystem enables targeted delivery to glioblastoma, followed by sequential release of siRNA and chemotherapeutic drug [151] *Copyright © 2025 Elsevier B.V*. (B) Dual nucleic acid co-delivery*:* Formation of the InCasApt theranostic platform, in which amine-functionalized dendritic MSNs (DMSN–NH_2_) are co-loaded with Cas13a/crRNA complexes, a DN-binding RNA aptamer precursor (DNBApt), a hairpin reporter (HR), and a caged photosensitizer (Ce6-DN). This multi-input construct integrates two nucleic acid modalities to achieve biomarker-activated diagnostic and therapeutic responses [152]. *Copyright © 2025 American Chemical Society.*

Early systems co-delivering siRNA with doxorubicin, paclitaxel, cisplatin, camptothecin or 5-fluorouracil demonstrated robust synergistic cytotoxicity [[144], [145], [146], [147], [148]]. More advanced designs include folic acid–targeted, lipid-encapsulated hollow MSNs co-loading paclitaxel and 5-fluorouracil for enhanced breast cancer therapy [149]; organosilica hybrid MSNs with tailored hydrophobicity for simultaneous nucleic acid and drug delivery [150]; and peptide–aptamer–capped MSNs loaded with temozolomide for dual targeting and synergistic cytotoxicity in glioblastoma [151] (Fig. 6A). Recent melanoma studies using oligonucleotide-based prodrugs further illustrate how tumour-specific targeting can be integrated to enhance microenvironment selectivity [25]. Overall, nucleic acid–drug combinations offer broader therapeutic applicability and stronger cytotoxic synergy than multi–nucleic-acid strategies, albeit at the cost of stricter control over release profiles due to cargo heterogeneity [146,153].

#### 4.2.2 Co-delivery of multiple nucleic acids

A second class of strategies focuses on the co-delivery of multiple nucleic acid modalities, motivated by the fact that many oncogenic pathways are co-regulated by several RNA species. Combining siRNA, miRNA or CRISPR systems enables multilayered and potentially programmable gene modulation, making this approach particularly relevant for tumors with complex transcriptional dysregulation or redundant signalling networks [147]. MSNs engineered with endosomolytic or tumour-penetrating functionalities can enhance cytosolic delivery and deep tumour penetration, supporting the precise pathway modulation required for multi-RNA therapies [154].

Beyond RNA-based combinations, silica platforms have also enabled the co-encapsulation of multiple plasmid DNAs, allowing simultaneous or temporally staggered expression of distinct genes from a single carrier. By exploiting hierarchical loading or spatial compartmentalization within MSNs, these systems can support sequential gene expression, mimicking viral-like genetic cascades and expanding the scope of programmable gene regulation [26].

This increased specificity, however, introduces additional challenges, including competitive loading between nucleic acids, the need for orthogonal release profiles, and preservation of the structural and functional integrity of CRISPR components. Representative examples include endosomolytic MSNs co-delivering siRNA and miRNA to achieve complementary pathway regulation [154], as well as more sophisticated constructs integrating CRISPR/Cas13 with RNA aptamers, fluorescent reporters and caged photosensitizers for biomarker-triggered diagnosis and on-demand therapeutic activation in tumour cells overexpressing miR-155 and miR-21 [152] (Fig. 6B). Compared with nucleic acid–drug systems, multi–nucleic-acid combinations offer greater mechanistic precision and programmability, but require more complex stabilization and loading strategies.

#### 4.2.3 Theranostic integration

The third major strategy, theranostic integration, embeds diagnostic elements directly within therapeutic MSNs to enable real-time monitoring of biodistribution, cargo release and biomarker expression [155]. This capability supports image-guided therapy, biomarker-triggered activation and longitudinal assessment of therapeutic responses, aligning with the broader movement toward personalized and adaptive treatment paradigms [133]. Silica's intrinsic optical transparency and chemical stability facilitate the incorporation of fluorescent dyes, quantum dots or SERS-active components, enabling high-resolution imaging without compromising structural integrity [156]. When combined with pH-, redox- or externally triggered gates, these platforms provide precise spatiotemporal control over therapeutic release [76].

Despite these advantages, integrating diagnostic and therapeutic functions increases design complexity, raising challenges such as signal interference, long-term *in vivo* stability and more demanding regulatory requirements [133]. Representative systems include fluorescent MSNs for real-time tracking of biodistribution and release kinetics, SERS-active silica platforms for molecular-level diagnostics, and CRISPR-based theranostic constructs coupling gene editing with optical reporters for biomarker-responsive activation [152]. Compared with purely therapeutic combinations, theranostic MSNs add a diagnostic dimension that enhances monitoring and control, albeit at the cost of increased engineering and regulatory complexity.

## 5 Conclusions and future perspectives: the roadmap to clinical translation

Taken together, the evidence discussed in this review supports the classification of silica-based nanocarriers as advanced platforms for nucleic acid delivery. In contrast to lipid and polymeric vectors, whose performance is often constrained by intrinsic coupling between structural stability, cargo loading and interfacial bioactivity, silica architectures decouple these parameters through a rigid yet chemically programmable scaffold [119,[157], [158], [159]]. This enables independent optimization of cargo protection, loading capacity, release kinetics, targeting and biodegradability within a single system. Compared to lipid nanoparticles, silica-based platforms offer superior structural resilience and expanded capacity for large and multicomponent genetic cargos, while tolerating modular hybridization without compromising integrity or reproducibility [[157], [158], [159], [160], [161]]. Relative to polymeric carriers, silica systems mitigate the classical polycation dilemma by confining buffering and release mechanisms within the framework rather than relying on excessive surface charge [162]. Together, this combination of mechanical robustness, architectural tunability and interfacial modularity underpins the designation of silica nanocarriers as advanced platforms for next-generation gene therapeutics, while also delineating the key engineering challenges that must be addressed to achieve clinical translation [133,163]. However, the same design complexity that underpins their versatility also introduces challenges in reproducibility, scalability and regulatory standardization.

### 5.1 Remaining design challenges

Despite significant progress in the design of silica nanocarriers, their clinical translation remains closely linked to challenges in scalable, reproducible and good manufacturing practice (GMP)-compliant manufacturing. Conventional laboratory syntheses often rely on poorly controlled parameters, such as batch mixing, template removal and surface functionalization, which can lead to batch-to-batch variability and hinder regulatory approval [164]. Recent advances in bioinspired and sol–gel–based manufacturing have begun to address these limitations by enabling aqueous, low-temperature, and energy-efficient synthesis routes that are more amenable to scale-up and process standardization [[165], [166], [167]]. These approaches improved reproducibility while reducing reliance on harsh conditions incompatible with nucleic acid stability. Nevertheless, further integration of in-line quality control, robust purification strategies and standardized surface chemistries will be required to meet the consistency, safety and documentation demands of clinical-grade nucleic acid formulations.

Beyond manufacturing constraints, MSNs face additional hurdles that must be addressed to enable widespread clinical adoption. First, scale-up and reproducibility remain problematic due to batch-to-batch variability and sensitivity to reaction conditions [133]. Second, standardized *in vivo* toxicity assessments are lacking, with inconsistent protocols across studies hindering regulatory approval and comparative analysis [168]. Third, high-throughput screening of SNP libraries is still underdeveloped, limiting the ability to rapidly identify optimal formulations for specific genetic payloads [133]. Additional challenges include long-term biodegradability, immune clearance, and the integration of predictive modeling to guide nanoparticle design [169].

The intrinsic immunological effects of silica nanocarriers also require careful consideration, particularly under repeated dosing regimens. Although activation of innate immune pathways, including complement, inflammasome signaling and cytokine release, has been reported for certain formulations, these responses are strongly dependent on particle size, porosity, surface chemistry and exposure conditions, and are most pronounced at high or repeated doses [170]. Systematic toxicological analyses indicate that amorphous and mesoporous silica nanoparticles, particularly when appropriately surface-engineered, generally exhibit low immunotoxicity at clinically relevant dose ranges [171,172]. In summary, immunological responses are strongly dependent on physicochemical properties and dose, with low immunotoxicity observed at exposure levels relevant for intravenous nanomedicine applications [173].

In addition to biological and regulatory considerations, storage stability and shelf-life represent practical challenges for the clinical translation of silica–nucleic acid formulations. Several studies have demonstrated that mesoporous and colloidal silica nanoparticles can preserve colloidal stability and nucleic acid functionality following freeze-drying when appropriate cryoprotectants and buffers are employed [174,175]. Lyophilized silica-based systems loaded with siRNA or miRNA retain transfection efficiency and biological activity for months when stored at refrigerated or frozen conditions, and in some cases even at room temperature [176]. Curiously, recent advances have demonstrated the exceptional ability of silica frameworks to protect nucleic acids from physical, chemical and biological degradation [26]. However, systematic studies addressing long-term shelf-life, large-scale reproducibility, and stability under clinically relevant storage and transport conditions remain limited, highlighting the need for further development toward standardized, commercially viable formulations.

### 5.2 Future hybrid platforms

To address these limitations, future research is expected to focus on hybrid systems that combine the structural advantages of silica with the biological performance of other materials. Lipid–silica hybrid nanoparticles (e.g., LC-MSNs) offer enhanced transfection efficiency and endosomal escape while preserving silica's mechanical integrity [55]. Similarly, polymer-grafted MSNs can introduce stealth properties, responsive release, and improved biocompatibility through surface engineering [177]. These hybrid platforms are poised to bridge the gap between robust cargo protection and efficient intracellular delivery, paving the way for personalized and multifunctional gene therapies.

In this context, silica-based platforms are particularly advantageous in scenarios where conventional lipid or polymeric vectors reach intrinsic limitations. These include the delivery of large or structurally complex cargos, such as long mRNA, plasmid DNA, or CRISPR/Cas ribonucleoproteins, where rigid frameworks and tunable pore architectures enable volumetric accommodation and cargo protection that are difficult to achieve with soft-matter systems. Silica nanocarriers are also especially well-suited for applications requiring long-term stability, tolerance to harsh processing or storage conditions, and precise control over release kinetics, as well as for modular or multifunctional formulations combining nucleic acids with drugs or diagnostic elements. In such settings, the mechanical robustness and architectural programmability of silica platforms provide capabilities that are not readily replicated by lipid- or polymer-based carriers.

To contextualize the potential of emerging hybrid systems, Table 5 provides a comparative assessment of key nucleic acid delivery platforms, including silica-based, lipid-based, and polymeric nanoparticles. The table outlines critical performance metrics such as cargo capacity, stability, endosomal escape efficiency, targeting specificity, and clinical readiness. This synthesis highlights the unique strengths and limitations of each system, reinforcing the rationale for hybridization strategies that integrate complementary functionalities to meet the demands of next-generation gene therapies.

**Table 5:** Comparative performance of nucleic acid delivery platforms.

Metric	Silica Nanoparticles (SNPs)	Lipid Nanoparticles (LNPs)	Polymeric Nanoparticles	Ref.
Cargo size capacity	High (up to CRISPR/Cas RNPs and plasmids)	Moderate (limited for large mRNA or RNPs)	Variable; often limited for bulky constructs	[[157], [158], [159]]
Stability	Excellent (rigid, non-leaky, resistant to degradation)	Moderate (prone to aggregation and enzymatic degradation)	Moderate to good; depends on polymer type and formulation	[102,119]
Endosomal escape	Tunable via buffering or redox-responsive designs	Good via ionizable lipids and fusogenicity	Often requires polycationic components (e.g., PEI)	[162,178,179]
Targeting specificity	High (via surface ligands, aptamers, membrane cloaking)	Moderate; limited ligand density due to lipid fluidity	Good; flexible ligand conjugation	[[180], [181], [182]]
Clinical readiness	Emerging; strong preclinical data, limited clinical trials	Approved for mRNA vaccines; scalable and regulatory precedent	Moderate; some approved systems, but toxicity remains a concern	[133,163]
Transfection efficiency	High; functionalization- and cell type–dependent	High; driven by ionizable lipids and membrane fusion	Moderate to high; limited by cytotoxicity and endosomal escape	[25,26,[183], [184], [185]]
Loading capacity	High; supports large and complex cargos	Moderate; optimized for mRNA/siRNA	Variable; polymer- and formulation-dependent	[160,161]

### 5.3 Clinical and regulatory landscape of silica-based gene delivery

Although silica nanoparticles have a long-standing record of use as excipients in pharmaceutical and food products, their application as systemic nanocarriers, particularly for nucleic acid delivery, has not yet reached formal regulatory approval. Several clinical studies with solid silica-based systems have nonetheless demonstrated encouraging safety and pharmacokinetic profiles, including oral trials in which drugs such as fenofibrate, ibuprofen and simvastatin formulated in silica or silica–lipid hybrid carriers showed improved bioavailability and were well tolerated in healthy volunteers, as well as cardiovascular and oncological applications using gold–silica nanoshells for photothermal ablation [168]. In parallel, ultrasmall silica platforms such as Cornell dots have progressed to phase I/II imaging trials in patients with metastatic melanoma and malignant brain tumors, with no particle-related adverse effects and rapid renal clearance [98]. By contrast, only a proof-of-concept data exists for mesoporous silica nanoparticles in humans, and no silica-based platform has yet entered clinical testing as a primary vector for genetic therapeutics. Bridging this translational gap will require robust evidence of controlled biodegradation to excretable species, harmonized GMP-compliant manufacturing and long-term safety under repeated dosing, while regulatory assessment must simultaneously address both the nanocarrier and the nucleic acid payload.

Continued integration of biodegradable framework design, immune-neutral surface engineering and scalable synthesis strategies is therefore essential to position silica architectures as clinically viable candidates for next-generation gene delivery. Silica nanocarriers are evolving from descriptive proof-of-concept systems into problem-driven, translational platforms. In fact, the problem-driven design strategies discussed in this review also align with key clinical translation requirements. Hybrid silica-based systems decouple cargo protection from interfacial bioactivity, facilitating reproducible manufacturing and batch-to-batch consistency, critical for regulatory evaluation. Stimuli-responsive and degradable architectures enable predictable release profiles and controlled biodegradation, supporting standardized dosing regimens and repeated administration. Moreover, the modularity of silica nanocarriers allows surface and targeting optimization without altering the core scaffold, in line with quality-by-design principles increasingly emphasized in nanomedicine development. Together, these features clarify how rational silica nanoarchitectures can be directly mapped onto a translational roadmap from design to clinical implementation.

Their unique combination of mechanical resilience, tunable porosity, and versatile surface chemistry positions them as strong contenders for next-generation nucleic acid therapeutics. Future progress will depend on harmonizing scaffold design with regulatory standards, ensuring biodegradability and immune neutrality, and integrating multifunctional capabilities for complex genetic payloads. By bridging nanoarchitectural innovation with clinical translation, silica nanoparticles may ultimately redefine the therapeutic landscape of gene therapy.

## CRediT authorship contribution statement

**Mónica L. Fanarraga:** Conceptualization, Writing – original draft. **Lorena García Hevia:** Conceptualization, Writing – original draft.

## Declaration of competing interest

The authors declare that they have no known competing financial interests or personal relationships that could have appeared to influence the work reported in this paper.

## Data Availability

No data was used for the research described in the article.
